# Gender-neutral human papillomavirus vaccination: an equitable and cost-effective public health investment

**DOI:** 10.3389/fpubh.2025.1725127

**Published:** 2026-01-05

**Authors:** Qingjing Luo, Qiang Ni

**Affiliations:** 1 West China Second University Hospital, Sichuan University, Chengdu, China; 2 Key Laboratory of Birth Defects and Related Diseases of Women and Children, Sichuan University, Ministry of Education, Chengdu, China

**Keywords:** equity monitoring, gender-neutral vaccination, HPV, immunization policy, school-based delivery

## Abstract

Human papillomavirus (HPV) drives a substantial cancer burden across all genders. This perspective argues that gender-neutral HPV vaccination is an equitable and cost-effective public health investment when designed for scale. Rather than presenting methods in detail, we synthesize recent epidemiology, policy positions, and economic evaluations to propose a simplicity-first pathway—a pragmatic model that, where permitted, prioritizes single-dose schedules, school-based delivery for adolescents aged 9–14, small, computable set of key performance indicators (KPIs) set (coverage, timeliness, series completion, and equity gaps), routine adverse events following immunization (AEFI) monitoring, and phased expansion as system capacity grows. The simplicity-first pathway advances global equity by protecting boys and girls alike, reducing stigma, and delivering both direct and indirect benefits to underserved groups, including sexual minority populations. In resource-constrained settings, single-dose policies (where adopted), integration with existing school-based platforms, and pooled multi-year procurement can reduce delivery complexity and improve value for money. Cost-effectiveness remains context-specific and is sensitive to vaccine price, achievable coverage, and which outcomes are included in the analysis. Policy implications: Countries should strongly consider gender-neutral vaccination as the default program design to accelerate population-level protection and close avoidable equity gaps, while adapting schedules and financing to national regulations, fiscal space, and information-system readiness. This simplicity-first framing offers a practical bridge between policy intent and sustainable routine implementation.

## 1 Introduction

Human papillomavirus (HPV) causes a substantial cancer burden in men as well as women. In 2022, approximately 95,000 male cancers were attributable to HPV worldwide, spanning oropharyngeal, anal, and penile sites; the distribution is heterogeneous across regions and income levels. In many low- and middle-income countries (LMICs), notably parts of South America and Sub-Saharan Africa—penile cancer accounts for a disproportionate share of male HPV-attributable cancers, while anal cancer burden is concentrated in key risk groups and remains under-measured outside high-income settings (1, 2). These epidemiologic patterns indicate that girls-only vaccination leaves important male risks insufficiently addressed across diverse contexts.

Many national HPV immunization programs still target girls only, particularly where introduction has been framed primarily around cervical cancer prevention or where supply and delivery capacity constrain expansion. For example, several Gavi-supported introductions prioritize a single cohort of girls aged 9–14 years, and established school-based programs such as Malaysia’s national HPV program have historically been implemented for schoolgirls. In such settings, reliance on herd protection from female vaccination depends on consistently high and equitable coverage; where coverage is heterogeneous or suboptimal, susceptible male cohorts can sustain transmission and remain insufficiently protected (3–5).

Our position therefore rests on primary epidemiology rather than policy: in settings with a meaningful male burden or substantial equity gaps, gender-neutral vaccination can close avoidable protection gaps. Our proposed “simplicity-first” pathway is grounded in both evidence and feasibility. WHO updated its recommendations to allow a single-dose HPV schedule for most girls and young women aged 9–20 years (with additional doses for selected groups, such as immunocompromised individuals), reflecting accumulating randomized and long-term follow-up evidence that one dose can provide high protection comparable to two doses against vaccine-type HPV infection. More recent noninferiority evidence further supports policy confidence in one-dose schedules. Programmatically, single-dose delivery reduces commodity demand, cold-chain and session time, and the operational risk of dose-2 attrition, constraints that are frequently cited as bottlenecks for rapid scale-up (6–9). In routine programs, initiation commonly exceeds series completion, indicating that multi-dose schedules can create additional opportunities for drop-off; this strengthens the practical case for dose-sparing strategies when supported by effectiveness evidence (10, 11). This perspective synthesizes recent epidemiology, program experience, and economic evaluations to propose a simplicity-first pathway to gender-neutral vaccination. The pathway prioritizes single-dose policies where permitted, school-based delivery for ages 9–14, and a lean, computable KPI set, while reserving operational details for subsequent sections (1).

## 2 Methods

This article is a policy-oriented perspective rather than a systematic review. We conducted a targeted narrative search of peer-reviewed and policy literature up to 25 October 2025 on: (i) HPV vaccine schedules (including one-dose policies), (ii) gender-neutral vaccination and priority groups, (iii) implementation strategies (delivery platforms, monitoring, safety surveillance), and (iv) economic and budgeting considerations. We searched PubMed/MEDLINE using combinations of keywords such as “HPV vaccine,” “single dose,” “one dose,” “gender-neutral,” “boys,” “school-based delivery,” “coverage,” “completion,” “dropout,” “AEFI,” and “cost-effectiveness.” We complemented the academic literature with key policy and market documents, including procurement and price resources from UNICEF and the PAHO Revolving Fund, and WHO position papers and guidance on HPV vaccination schedules and cervical cancer elimination (6, 12, 13). We prioritized guidance documents, systematic reviews, and empirical studies with clear implications for program design, and synthesized evidence narratively to derive a practical implementation pathway and a minimum KPI package for routine performance management.

## 3 Where we are now: evidence and policy status

### 3.1 Policy adoption and global coverage

Policy has moved faster than delivery: supply constraints, school-session capacity, and sex-disaggregated monitoring often determine whether gender-neutral expansion is feasible. The WHO’s 2022 position allows program flexibility, including a one-dose schedule, and notes the potential value of gender-neutral vaccination when supported by local epidemiology and economics (13). Early adopters (Australia, the United Kingdom, Nordic countries, Canada, New Zealand) run gender-neutral, school-based programs with high uptake and systematic catch-up (14). Yet coverage remains uneven: in 2024 only about 31% of eligible girls received at least one dose worldwide, with large regional and income-level gaps, underscoring that current delivery models are insufficient for equitable protection (14).

### 3.2 Generalizability to LMICs

Real-world impact is evident beyond high-income settings. Rwanda and Bhutan achieved very high coverage (≥90 percent) with school-based delivery and documented declines in vaccine-type HPV. Malaysia and Brazil have reported recovery or improvement in uptake after the pandemic and declines in genital warts and cervical precancer following national rollout (13–17). Across these settings, once adolescent coverage approaches about 70% or higher, early outcome gains emerge, provided delivery platforms are reliable and monitoring is transparent. Table 1 summarizes representative programs across income levels, including policy design, delivery platform, recent coverage, and early outcome signals (15–22).

**Table 1:** Comparative overview of HPV vaccination programs (policy, coverage, early outcomes).

Country	Income level	Policy (start year)	Delivery	Latest coverage (girls/boys, year)	Observed outcome (yrs. post-start)
United Kingdom	High	Gender-neutral (2019)	School	75%/70% (2024)	Large declines in vaccine-type HPV; program inequalities persist regionally (18).
Sweden	High	Gender-neutral (2020)	School	91%/87% (2024)	Genital warts ↓ 50–90% in young cohorts; continued declines in both sexes (19).
Australia	High	Gender-neutral (2013)	School	73%/70% (2024)	Genital warts ↓ ≈ 70–90% in target ages; fewer treatments/hospitalizations (20).
Rwanda	Low-Middle	Girls first (2011; high coverage)	School→routine	NR/77% (2024)	Vaccine-type HPV ↓; sustained national coverage 2011–2018 (15).
Bhutan	Low-Middle	Gender-neutral (2020)	School	~90–96%	High coverage sustained; early impact consistent with high-coverage settings (16).
Malaysia	Upper-Middle	Girls-only (2010)	School	78%/NR (2024)	HPV prevalence improvements in younger women (17).
Argentina	Upper-Middle	Boys added (2017)	Mixed	55%/51% (2024)	HPV16/18 ↓ > 90% among vaccinated girls; male impact emerging (21).
Brazil	Upper-Middle	Boys added (2017)	Mixed	79%/65% (2024)	↓ hospitalizations for anogenital warts and high-grade CIN (22).

Income level follows the World Bank classification. “Policy (start year)” is the year of national introduction for the listed policy; “Delivery” is the primary platform. “Latest coverage” reports the most recent officially published coverage for the routine adolescent target group and is shown as girls%/boys% (reporting year); NR indicates not reported for that sex/year; ranges reflect reported intervals. “Observed outcome” summarizes ecological/observational signals at ~3–10 years post-start from the cited sources; arrows indicate direction of change. Mixed = school plus primary health care (PHC). CIN, cervical intraepithelial neoplasia. Figures are not strictly comparable across countries due to differences in age bands, denominators, reporting years, and data sources [see refs (15–22)].

### 3.3 Regional feasibility and political-economy barriers

Pre-specify consent pathways, a misinformation response protocol, and a minimum mop-up cadence; otherwise localized hesitancy can stall uptake even with endorsed policy. In Japan, suspension of proactive recommendation (2013–2022) caused a collapse in uptake; hesitancy persists despite resumption, and male vaccination, though authorized, is not universally publicly funded. In many African and Asian contexts, parental-consent requirements, moral framing of adolescent sexuality, and misinformation constrain uptake even where school delivery is feasible. These dynamics help explain heterogeneous herd effects and support sex-neutral schedules as a structural buffer where coverage is fragile or variable (23–26).

### 3.4 Modeling implications and transition pathways

Dynamic transmission models indicate that adding boys accelerates and stabilizes declines in HPV16 and related outcomes under moderate or heterogeneous coverage, including one-dose schedules (13, 14). A low-disruption pathway is phased: deliver girls first via schools; expand to gender-neutral cohorts only after ≥2 terms without stockouts and sustained dose-1 coverage ≥70–80%; then add targeted catch-up for MSM/out-of-school. Where coverage plateaus below about 80% or inequities persist, models support earlier gender-neutral adoption to secure durable, population-level protection (13, 14).

## 4 Public-health rationale for vaccinating boys

Girls-only programs leave gaps in protection—for boys, men who have sex with men, and adolescents with limited-service access. We therefore proceed to equity and priority populations (Section 4.1) and then quantify the cost-effectiveness of broadening eligibility (Section 4.2).

### 4.1 Direct benefits and transmission dynamics

HPV causes substantial disease in males—including oropharyngeal, anal, and penile cancers and anogenital warts—and men sustain transmission across heterosexual and same-sex networks. Penile cancer incidence is highest in parts of South America and Sub-Saharan Africa, underscoring the need for direct male protection beyond high-income settings (27, 28). Vaccinating boys provides individual protection and, where female coverage is suboptimal (e.g., <80%), helps close residual transmission loops maintained by susceptible male cohorts. Population-level syntheses show strong herd effects, with large declines in vaccine-type HPV and anogenital warts under multi-cohort or high-coverage programs (29). Real-world gender-neutral programs report reductions in anal, penile, and oral vaccine-type HPV among young men, including MSM, after school-based implementation (30). Over longer horizons, age–period–cohort analyses suggest corresponding declines in male oropharyngeal cancer as vaccinated cohorts age (31). In short, gender-neutral vaccination addresses both direct male burden and the network dynamics that limit the robustness of girls-only strategies.

### 4.2 Equity and priority populations (alignment with WHO/Immunization Agenda 2030)

MSM and other underserved male groups (e.g., migrants, out-of-school youth) gain little herd protection from girls-only models because their exposure pathways are only partially—or not at all—buffered by female vaccination; several programs therefore introduced targeted MSM vaccination alongside, or as a precursor to, broader gender-neutral strategies (32). Gender-neutral vaccination reduces disparities by ensuring individual protection regardless of partner patterns while lowering overall circulation, aligning with Immunization Agenda 2030’s commitment to “leave no one behind” (33). Programmatic flexibility—including a one-dose schedule for most 9–20-year-olds, where permitted—further improves affordability and reach without undermining elimination goals (13).

## 5 Economic rationale and value for money

### 5.1 Cost-effectiveness under real program conditions

Dynamic transmission evaluations that reflect real delivery conditions (imperfect coverage, mixed sexual networks, herd effects, and the prevention of cancers and warts across genders) consistently suggest that expanding protection beyond girls-only strategies (including gender-neutral vaccination and, where modeled, dose-sparing one-dose policies) offers better value for money (34–37). Models that include dose-sparing (one-dose) policies and declining vaccine prices generally produce more favorable incremental cost-effectiveness ratios (ICERs) and, in some settings, long-run cost-saving when broader outcomes (oropharyngeal, anal, penile cancers; anogenital warts) are counted (13, 38, 39). This matches public-health logic: vaccinating boys accelerates transmission decline and directly prevents male cancers and warts, bolstering resilience where female coverage is suboptimal or disrupted (29, 40).

Representative modeled results (summarized in Supplementary Table S1) indicate that gender-neutral strategies are generally cost-effective, or even cost-saving, when vaccine prices are moderated and broader outcomes (including male oropharyngeal, anal, and penile cancers and anogenital warts) are counted (34, 38, 41, 42). Across settings, ICERs are most sensitive to assumptions about vaccine price, achievable coverage (especially for boys), dose durability, discount rates, and the time horizon used.

### 5.2 Budget impact, delivery overhead, and LMIC affordability

Expanding from girls-only to GNV typically adds limited marginal delivery overhead when existing school-based platforms are used (shared rosters, consent, routes, AEFI workflows). Delivery-cost studies in LMICs report wide but generally modest non-commodity costs per dose, with several school programs near the lower end (e.g., Rwanda ≈US$1.03 financial/US$3.09 economic per dose, excluding vaccine/supplies), supporting the reality that commodities dominate incremental costs at scale (43, 44).

Affordability improves when dose-sparing policies are tied to a 3–5-year budget forecast (cohort × wastage × buffer) and procured via pooled mechanisms; the Pan American Health Organization and the United Nations Children’s Fund reference prices can serve as a conservative budgeting ceiling. WHO’s 2022 position endorses single-dose HPV vaccination for most 9–20-year-olds (except immunocompromised), and in October 2024 WHO prequalified Cecolin^®^ for single-dose use, effectively halving commodity needs and simplifying logistics; service-cost modeling likewise shows that moving from two doses to one nearly halves the cost per fully vaccinated adolescent (excluding vaccine price) (45, 46). Using pooled procurement reference prices (e.g., Pan American Health Organization/United Nations Children’s Fund), ministries can set a conservative “price ceiling” for budget impact analysis, negotiate multi-year volume commitments, and transparently communicate affordability scenarios (base/low/high coverage), making expansion decisions less sensitive to short-term price shocks (47, 48). In parallel, Gavi co-financing phases in domestic cost-sharing while preserving pooled prices, offering a glidepath to scale within constrained fiscal space (49, 50).

Practical takeaway. In most LMIC and upper-middle-income settings, the incremental fiscal signal of adding boys is commodity-driven, not delivery-driven, when school platforms exist; pairing one-dose schedules, pooled procurement, and predictable co-financing keeps expansion both affordable and plannable over multi-year horizons (see Supplementary Table S2) (41, 47–51).

### 5.3 Returns on investment and fiscal resilience

Over the medium–long term, GNV yields strong return on investment by averting treatment costs and productivity losses across cervical and non-cervical disease. In Latin America, Colombia’s analysis found GNV cost-saving with US$ 88–184 million in net savings over 100 years; in Canada, adding males generated Canadian dollar 8–28 million in net savings for a single cohort under plausible assumptions (39, 52). Because school-based delivery has large, fixed components, the marginal cost of adding boys is chiefly vaccines and syringes—contained through one-dose policies, pooled procurement, and staged co-financing using transparent reference prices from United Nations Children’s Fund and Pan American Health Organization (47, 48). Together, these levers support short-run affordability and long-run fiscal resilience, locking in durable, gender-inclusive cancer prevention with predictable recurrent costs.

## 6 Barriers to adoption and how to overcome them

### 6.1 Demand-side barriers and message reframing

Adopting gender-neutral HPV vaccination faces reinforcing barriers: the notion that HPV is a “women’s issue,” budgets focused narrowly on cervical outcomes, hesitancy driven by online misinformation and stigma, and limited capacity for comprehensive school-based health education in conservative or resource-limited settings. A pragmatic start is evidence-based reframing anchored in cancer prevention for all: state the male burden (oropharyngeal, anal, penile cancers; anogenital warts) alongside cervical cancer, align with life-course immunization, avoid sexualized framing, and deploy short, pre-tested messages and frequently asked questions that address local myths and practical concerns (side effects, fertility, eligibility, cost) (53–55).

### 6.2 Trusted messengers and behavioral tactics

Reframed messages work best via trusted messengers (clinicians, teachers/school leaders, community and faith leaders, and youth influencers), paired with presumptive recommendations, simplified consent, and on-site school vaccination. Reinforce with immunization information system reminder–recall, opt-out defaults where lawful/ethical, and small nudges (e.g., same-day sessions tied to school events). Tailor materials for migrants and out-of-school youth (translated assets, text message and WhatsApp, peer navigators). Use a brief Behavioral and Social Drivers checklist (thinking/feeling, social processes, motivation, practical barriers) with parent–teacher groups to guide weekly micro-adjustments in outreach and delivery (54, 56, 57).

### 6.3 Supply-side fixes, normalization, and feasibility signals

Sustain confidence by matching communication with predictable supply and reliable service delivery and normalize HPV vaccination as a routine adolescent vaccine for all genders alongside tetanus–diphtheria–acellular pertussis and meningococcal. Strengthen safety surveillance: clarify reporting lines, train school vaccinators on case definitions/documentation, and publish regular safety briefs referencing the national AEFI system (e.g., Canada’s Canadian Adverse Events Following Immunization Surveillance System) (58). Feasibility signals from early school-based introductions (Rwanda and Bhutan achieved ≥90% coverage with sustained early impact) show that gender-neutral HPV vaccination is operationally feasible and socially acceptable when delivered through existing school platforms with simple consent and closed-loop data workflows (59–61).

## 7 Implementation playbook (minimum viable package first, then scale)

### 7.1 Minimum viable package: use existing adolescent platforms

To minimize marginal delivery cost, integrate boys into the same school-session roster, consent workflow, and cold-chain route as girls. The key constraint is not staff time per se but session reliability (missed days → dropouts), so programs should pre-plan at least one mop-up visit per term and use immunization information system-generated absentee/defaulter lists to target catch-up. This approach leverages existing adolescent vaccination platforms to move quickly while controlling costs (13, 56).

### 7.2 Tailoring by income level and cultural context

LMIC: Default to single dose per WHO (except immunocompromised); procure via pooled mechanisms (e.g., United Nations Children’s Fund); prioritize in-school sessions with light outreach; use translated, youth-friendly channels; monitor four KPIs only (dose-1, completion, timeliness, equity gap); publish quarterly safety briefs.

Upper-middle-income countries/high-income countries: Implement gender-neutral from the outset; retain school-based delivery; add closed-loop referral to primary care for absentees; run monthly dashboards with equity-stratified breakdowns (socioeconomic status/region/school type); consider targeted catch-up for MSM and out-of-school youth. This graduated approach aligns with Immunization Agenda 2030 equity/data-for-action principles and WHO schedule flexibility (13, 33, 47).

### 7.3 Monitoring and KPIs; phased scale-up

Use a traffic-light dashboard where each KPI has a predefined action: if dose-1 coverage is red, increase session density; if completion is red, add mop-up and reminder–recall; if timeliness is red, shorten the interval between baseline and mop-up; if the equity-gap index is red, deploy targeted outreach for the worst-performing subgroup rather than expanding geography (see Table 2) (62–64).

**Table 2:** Policy-facing KPI dashboard for weekly review.

KPI (policy name)	One-line operational definition	Traffic-light thresholds	If red, do next week	Equity stratifies
Dose 1 coverage	% of enrolled target-grade students receiving ≥1 valid HPV dose this term	Red <65% • Amber 65–79% • Green ≥80%	Red/Amber: add 1 extra school session; run IIS reminder SMS; retrain consent collectors	Sex, district/region, urban–rural, school type, SES quintile
Series completion	% of dose-1 recipients who meet national completion rule within 12 months (single-dose or 2-dose with min interval)	Red <70% • Amber 70–84% • Green ≥85%	Export defaulter list from IIS; schedule catch-up day; call school principals to call absentees	Sex, district/region, SES
Timeliness	% of doses administered within planned session date ±30 days	Red <70% • Amber 70–79% • Green ≥80%	Shift late schools into earlier routes; increase on-site vaccinators on high-enrolment days	Sex, district, school type
Equity-gap index	Coverage ratio (lowest ÷ highest SES quintile) + absolute pp gap	Red ratio <0.90 or gap >10 pp • Amber 0.90–0.94 or 6–10 pp • Green ≥0.95 and ≤5 pp	Deploy targeted outreach at bottom-quintile schools; translated materials; peer educator slots	SES quintile (mandatory); add migrant/out-of-school if available

IIS, Immunization Information System; AEFI, adverse event following immunization; SES, socioeconomic status; pp, percentage points. Minimal data sources: school rosters; session calendar; IIS extracts; AEFI register. Review cadence: weekly at district, monthly national. Alignment: WHO BeSD, IA2030 (equity/data-for-action), and WHO 2022 HPV schedule flexibility (single dose where permitted) (62–64). Thresholds are illustrative and should be adapted to national baselines.

Pilot (90 days): In 4–6 schools, test a gender-neutral, single-dose (where permitted), school-based bundle: presumptive scripts, trusted messengers, translated consent, reminder/recall, and one mop-up session, with cultural tailoring. Success is defined by the Table 2 KPIs (Dose-1 ≥ 80%, completion ≥85%, timeliness ≥85%, equity gap ≤5 pp) and complete AEFI reporting; otherwise run one Plan–Do–Study–Act targeting the worst subgroup.

### 7.4 Procurement and affordability

Pair single-dose schedules with pooled procurement and multi-year agreements to sustain affordability. Plan with transparent reference prices; as volumes rise and prices fall, value for money improves. Maintain stakeholder confidence, especially among finance and education ministries, through short quarterly briefs on performance and safety (13, 47).

### 7.5 District-level KPI checklist for routine reviews

To make performance management workable without advanced analytics, we recommend a minimum viable KPI checklist that can be reviewed in 10–15 min at district level. The checklist is presented as Table 2 and includes three elements for each metric: (i) a one-line operational definition, (ii) a simple threshold for traffic-light status, and (iii) a predefined corrective action that must be triggered within 1 week when the metric turns “amber” or “red.” At a minimum, routine review should cover: (1) dose-1 coverage (sex-disaggregated), (2) on-time completion (or on-time dose-1 where one-dose schedules are used), (3) dropout between invitation and vaccination sessions, (4) equity gaps (by sex, district, and school type), and (5) safety signal completeness (AEFI reporting timeliness and case follow-up). Full calculation logic and data rules are provided in Supplementary Methods S1, S2, but Table 2 is sufficient for routine operational decision-making.

## 8 Discussion and conclusion

This perspective argues that gender-neutral HPV vaccination is not simply an “add-on” to girls-only programs but a pragmatic strategy to reduce avoidable male cancer burden and equity gaps when transmission dynamics and coverage patterns make herd protection incomplete. The proposed pathway emphasizes operational simplicity: dose simplification where permitted, school-based delivery for ages 9–14, and a minimum, computable KPI set, because implementation friction, rather than intent, is often the binding constraint on coverage and sustainability.

A key policy implication is that program design should be evaluated against three practical questions: (i) can the delivery platform reliably reach adolescents with high and equitable coverage, (ii) does the schedule minimize avoidable attrition points (especially dose-2 drop-off), and (iii) can performance and safety be monitored with routine data systems at district level. Where these conditions are met, gender-neutral vaccination can improve resilience to coverage heterogeneity and reduce reliance on indirect protection for males, including groups that may be poorly served by girls-only strategies.

Rather than prescribing a single universal model, we propose a decision logic that aligns epidemiology, feasibility, and affordability. Countries can start with a minimum viable package, demonstrate early performance using a transparent dashboard, and scale stepwise with procurement planning and equity-focused monitoring.

### 8.1 Limitations

This synthesis relies primarily on secondary sources and modeling studies, which introduces several constraints.

(i) Assumptions vary across models (e.g., discount rates, vaccine price trajectories, durability of one-dose schedules, cervical screening background, case definitions), limiting direct comparability.
(ii) There is contextual uncertainty in many LMIC and upper-middle-income countries settings, where male cancer incidence, MSM-specific burden, and real-world delivery costs are less completely measured.
(iii) Many high-value endpoints unfold only over long-time horizons: key cancers in males (oropharyngeal and anal) emerge after long latency, so current projections necessarily rely on indirect outcomes and intermediate markers.

Program evaluation evidence is also subject to selection and information bias. Coverage denominators, sex and socioeconomic disaggregation, and AEFI reporting completeness differ across registries. Generalizability from early high-coverage adopters may overstate feasibility in settings where school-based delivery platforms are weaker or more fragmented. Finally, economic value is highly sensitive to vaccine price and dose durability. Many available models still lack full probabilistic uncertainty analyses and distributional (equity-weighted) outcomes, which means they may understate or obscure the equity impact of gender-neutral vaccination.

### 8.2 Future directions

First, pragmatic real-world evaluations are needed: Trials and quasi-experimental designs (e.g., stepped-wedge rollout, interrupted time series, difference-in-differences) embedded within school-based delivery should measure dose 1 uptake, on-time completion, dropout, and equity gaps, together with linked safety dashboards. Prospective registration and open analytic code would strengthen credibility and policy usefulness.

Second, long-term disease surveillance must be strengthened: Registry linkages should track male cancers (oropharyngeal, anal, penile) with HPV typing where feasible, while periodic oral and anal HPV prevalence surveys provide leading indicators as cancer endpoints mature. Standardized surveillance for MSM and out-of-school youth, groups that receive little indirect protection from girls-only programs and are often underrepresented in routine reports, would allow countries to quantify coverage gaps, monitor safety, and target catch-up strategies.

Third, economics under real constraints require better data: Prospective micro-costing of school-based delivery, with and without one-dose schedules, should be paired with threshold and scenario analyses for different vaccine products under pooled procurement. Distributional cost-effectiveness and return-on-investment analyses are needed to quantify equity gains and fiscal resilience, rather than relying solely on average incremental cost-effectiveness ratios.

## 9 Conclusion

In settings with meaningful male HPV-attributable cancer burden or substantial equity gaps, gender-neutral HPV vaccination can close avoidable protection gaps. A simplicity-first implementation pathway—supported by evidence for dose-sparing schedules where permitted, school-based delivery, and a minimum KPI dashboard—can help countries move quickly while maintaining accountability for coverage, equity, and safety. Future work should prioritize pragmatic effectiveness evaluations, surveillance linkages for male outcomes, and real-world costing under routine constraints to inform sustainable scale-up.

## Data Availability

The original contributions presented in the study are included in the article/Supplementary material, further inquiries can be directed to the corresponding author.

## References

[1] Zhang J Ke Y Chen C Jiang Z Liu H Liu Y . HPV cancer burden by anatomical site, country, and region in 2022. Sci Rep. (2025) 15:21048. doi: 10.1038/s41598-025-06700-8, 40593150 PMC12214776

[2] Fu L Tian T Yao K Chen X-F Luo G Gao Y . Global pattern and trends in penile cancer incidence: population-based study. JMIR Public Health Surveill. (2022) 8:e34874. doi: 10.2196/34874, 35793140 PMC9301560

[3] Murewanhema G Moyo E Dzobo M Mandishora-Dube RS Dzinamarira T. Human papilloma virus vaccination in the resource-limited settings of sub-Saharan Africa: challenges and recommendations. Vaccine X. (2024) 20:100549. Published 2024 Aug 20. doi: 10.1016/j.jvacx.2024.100549, 39263366 PMC11388769

[4] Bogaards JA Wallinga J Brakenhoff RH Meijer CJ Berkhof J. Direct benefit of vaccinating boys along with girls against oncogenic human papillomavirus: bayesian evidence synthesis. BMJ. (2015) 350:h2016. doi: 10.1136/bmj.h2016, 25985328 PMC4428278

[5] Ali H Donovan B Wand H Read TRH Regan DG Grulich AE . Genital warts in young Australians five years into national human papillomavirus vaccination programme: national surveillance data. BMJ. (2013) 346:f2032. Published 2013 Apr 18. doi: 10.1136/bmj.f2032, 23599298

[6] UNICEF. From evidence to impact: A synthesis of gender analyses in immunization. New York: UNICEF; 2024. Available online at: https://www.unicef.org/media/172671/file/Evidence-impact-gender-immunization-report.pdf (accessed Dec 2, 2025).

[7] Barnabas RV Brown ER Onono MA Bukusi EA Njoroge B Winer RL . Efficacy of single-dose HPV vaccination among young African women. NEJM Evid. (2022) 1:EVIDoa2100056. doi: 10.1056/EVIDoa2100056, 35693874 PMC9172784

[8] Kreimer AR Porras C Liu D Hildesheim A Carvajal LJ Ocampo R . Noninferiority of one HPV vaccine dose to two doses. N Engl J Med. [online ahead of print]. (2025). doi: 10.1056/NEJMoa2506765 41337735

[9] Barnabas RV Brown ER Onono MA Bukusi EA Njoroge B Winer RL . Durability of single-dose HPV vaccination in young Kenyan women: randomized controlled trial 3-year results. Nat Med. (2023) 29:3224–32. doi: 10.1038/s41591-023-02658-0, 38049621 PMC10719107

[10] Ilic I Ilic M. Human papillomavirus vaccination coverage estimates among the primary target cohort (9-14-year-old girls) in the world (2010-2024). Vaccine. (2025) 13:1010. doi: 10.3390/vaccines13101010, 41150398 PMC12568012

[11] World Health Organization. New HPV vaccines – Current landscape and future directions. HPV World; 2024. Available online at: https://www.hpvworld.com/articulos/new-hpv-vaccines-current-landscape-and-future-directions/ (accessed Dec 2, 2025)

[12] Pan American Health Organization (PAHO). PAHO revolving fund: Vaccine prices for the calendar year 2024. Washington (DC): PAHO; 2024. Available online at: https://www.paho.org/sites/default/files/2024-11/prices-vac-2024-eng-november.pdf (accessed Dec 2, 2025)

[13] World Health Organization. Human papillomavirus vaccines: WHO position paper, December 2022. 2022. Available online at: https://www.who.int/publications/i/item/who-wer9750-645-672 (accessed October 7, 2025).

[14] World Health Organization. Global childhood vaccination coverage holds steady, yet over 14 million infants remain unvaccinated — WHO, UNICEF (2025). Available online at: https://www.who.int/news/item/15-07-2025-global-childhood-vaccination-coverage-holds-steady-yet-over-14-million-infants-remain-unvaccinated-who-unicef (accessed October 8, 2025).

[15] Sayinzoga F Umulisa MC Sibomana H Tenet V Baussano I Clifford GM. Human papillomavirus vaccine coverage in Rwanda: A population-level analysis by birth cohort. Vaccine. (2020) 38:4001–5. doi: 10.1016/j.vaccine.2020.04.021, 32336599 PMC7221340

[16] Yangchen S Felsher M Acosta D Sukarom I Wu L Phuntsho S . Lessons learned from Bhutan on extending girls-only HPV vaccination program to boys: A qualitative study. Asia Pac J Public Health. (2024) 36:580–8. doi: 10.1177/10105395241273296, 39169479

[17] Khoo SP Muhammad Ridzuan Tan NA Rajasuriar R Nasir NH Gravitt P Ng CW . Changes in genital human papillomavirus (HPV) prevalence among urban females a decade after the Malaysian HPV vaccination program. PLoS One. (2022) 17:e0278477. doi: 10.1371/journal.pone.0278477, 36538522 PMC9767374

[18] Checchi M Mesher D Panwar K Anderson A Beddows S Soldan K. The impact of over ten years of HPV vaccination in England: surveillance of type-specific HPV in young sexually active females. Vaccine. (2023) 41:6734–44. doi: 10.1016/j.vaccine.2023.10.002, 37821315

[19] Astorga Alsina AM Herweijer E Lei J. Population-level impact of human papillomavirus vaccination on the incidence of genital warts in Sweden. J Infect Dis. (2025) 232:e54–63. doi: 10.1093/infdis/jiaf052, 39879631 PMC12308650

[20] Hsu TY Pavelyev A Saxena K Walia A Prabhu VS. Clinical and economic impact of school-based nonavalent human papillomavirus vaccination in females in Malaysia. Singapore Med J. [online ahead of print]. (2023). doi: 10.4103/singaporemedj.SMJ-2020-446 PMC13143302 38037773

[21] Bruni L Albero G Serrano B Mena M Collado JJ Gómez D . Human papillomavirus and related diseases in Argentina. Summary Report 10 March 2023. (2023). Available online at: https://hpvcentre.net/statistics/reports/ARG.pdf (accessed October 24, 2025).

[22] Bierrenbach AL Parellada CI Rocha Sarmento TT Barbour Oliveira JC Gonçalves Queijo R Orengo JC. Impact of HPV vaccination on the hospitalizations for anogenital warts and high-grade cervical intraepithelial neoplasia in Brazil: A national analysis. Hum Vaccin Immunother. (2025) 21:2514949. doi: 10.1080/21645515.2025.2514949, 40518563 PMC12169030

[23] Takahashi T Ichimiya M Tomono M Minoura R Kinoshita T Imanishi Y . Overcoming HPV vaccine hesitancy in Japan: a narrative review of safety evidence, risk communication, and policy approaches. Vaccine. (2025) 13:590. doi: 10.3390/vaccines13060590, 40573921 PMC12197371

[24] Yagi A Ueda Y Oka E Nakagawa S Kimura T Shimoya K. Even though active recommendation for HPV vaccination has restarted, Japan's rates have not recovered. Cancer Sci. (2024) 115:2410–6. doi: 10.1111/cas.16167, 38698561 PMC11247556

[25] Kaneda Y Namba M Gyeltshen T. A call for bridging gender gap in HPV vaccination policies in Japan. Health Sci Rep. (2023) 6:e1421. doi: 10.1002/hsr2.1421, 37441131 PMC10333723

[26] Kutz JM Rausche P Gheit T Puradiredja DI Fusco D. Barriers and facilitators of HPV vaccination in sub-saharan Africa: a systematic review. BMC Public Health. (2023) 23:974. doi: 10.1186/s12889-023-15842-1, 37237329 PMC10214362

[27] International Agency for Research on Cancer & World Health Organization. Penis cancer fact sheet (GLOBOCAN). Geneva: IARC/WHO; 2022. Available online at: https://www.gco.iarc.who.int/media/globocan/factsheets/cancers/26-penis-fact-sheet.pdf (accessed October 25, 2025).

[28] International Agency for Research on Cancer & World Health Organization. Sub-Saharan Africa fact sheet (GLOBOCAN). Geneva: IARC/WHO; 2022. Available online at: https://gco.iarc.who.int/media/globocan/factsheets/populations/963-sub-saharan-africa-fact-sheet.pdf (accessed October 25, 2025).

[29] Drolet M Bénard É Pérez N Brisson M HPV Vaccination Impact Study Group. Population-level impact and herd effects following the introduction of human papillomavirus vaccination programmes: updated systematic review and meta-analysis. Lancet. (2019) 394:497–509. doi: 10.1016/S0140-6736(19)30298-3, 31255301 PMC7316527

[30] Chow EPF Tabrizi SN Fairley CK Wigan R Machalek DA Garland SM . Prevalence of human papillomavirus in young men who have sex with men after the implementation of gender-neutral HPV vaccination: a repeated cross-sectional study. Lancet Infect Dis. (2021) 21:1448–57. doi: 10.1016/S1473-3099(20)30687-3, 34043963

[31] Cao C Lee A Kang JJ Yacoub I Zakeri K Dee EC . Updated estimates of patients with oropharyngeal cancer in the US. JAMA Netw Open. (2025) 8:e2539258. doi: 10.1001/jamanetworkopen.2025.39258, 41134572 PMC12552931

[32] Joint Committee on Vaccination and Immunisation (JCVI). Interim position statement on HPV vaccination of men who have sex with men (MSM). London: Department of Health & Social Care; 2013. Available online at: https://assets.publishing.service.gov.uk/media/5a7dac11ed915d2acb6ed73f/JCVI_interim_statement_HPV_vacc.pdf (accessed October 25, 2025).

[33] World Health Organization. Immunization agenda 2030: A global strategy to leave no one behind. Geneva: WHO; 2020. Available online at: https://www.who.int/docs/default-source/immunization/strategy/ia2030/ia2030-document-en.pdf (accessed October 25, 2025).

[34] Palmer C Matsuki T Tobe K You X Chen YT. Public health impact and cost-effectiveness of implementing gender-neutral vaccination with a 9-valent HPV vaccine in Japan: a modeling study. J Med Econ. (2025) 28:974–85. doi: 10.1080/13696998.2025.2520703, 40528568

[35] Daniels V Saxena K Patterson-Lomba O Gomez-Lievano A At Thobari J Durand N . Modeling the health and economic implications of adopting a 1-dose 9-valent human papillomavirus vaccination program in adolescents in low/middle-income countries: an analysis of Indonesia. PLoS One. (2024) 19:e0310591. doi: 10.1371/journal.pone.0310591, 39570910 PMC11581242

[36] Linertová R Guirado-Fuentes C Mar-Medina J Teljeur C. Cost-effectiveness and epidemiological impact of gender-neutral HPV vaccination in Spain. Hum Vaccin Immunother. (2022) 18:2127983. doi: 10.1080/21645515.2022.2127983, 36347243 PMC9746431

[37] Joint Committee on Vaccination and Immunisation. Statement on HPV vaccination. London: Department of Health and Social Care; 2018. Available online at: https://assets.publishing.service.gov.uk/media/5b4e0a34e5274a73119d7614/JCVI_Statement_on_HPV_vaccination_2018.pdf (accessed October 8, 2025).

[38] Wahab MT Tan RKJ Cook AR Prem K. Impact of including boys in the national school-based human papillomavirus vaccination programme in Singapore: A modelling-based cost-effectiveness analysis. Vaccine. (2023) 41:1934–42. doi: 10.1016/j.vaccine.2023.02.025, 36797100

[39] Carrasquilla M Zakzuk Sierra J Alvis-Zakzuk NJ de la Gomez Rosa F Beltran-Rodriguez C Rojas M . PIN9 public health and economic impact of a gender-neutral quadrivalent human papillomavirus vaccination program in Colombia. Value Health Reg Issues. (2019) 19:S41–2. doi: 10.1016/j.vhri.2019.08.240

[40] Pearson AL Kvizhinadze G Wilson N Smith M Canfell K Blakely T. Is expanding HPV vaccination programs to include school-aged boys likely to be value-for-money: a cost-utility analysis in a country with an existing school-girl program. BMC Infect Dis. (2014) 14:351. Published 2014 Jun 26. doi: 10.1186/1471-2334-14-351, 24965837 PMC4082618

[41] van Schalkwyk C Meyer-Rath G Masuku S Jamieson L Bloem P Rangaraj A . Cost-effectiveness of different HPV vaccination strategies for cervical cancer prevention in South Africa. Vaccine. (2025) 64:127770. doi: 10.1016/j.vaccine.2025.127770, 40992075

[42] Carrasquilla M Alvis-Zakzuk NJ Zakzuk Sierra J la Gómez De Rosa F Beltran C Rojas M . Estimating the impact of a gender-neutral quadrivalent human papillomavirus vaccination program in all HPV 6/11/16/18-related diseases in Colombia. Value Health. (2020) 23:S170–1. doi: 10.1016/j.jval.2020.04.489, 41377697

[43] Slavkovsky R Callen E Pecenka C Mvundura M. Costs of human papillomavirus vaccine delivery in low- and middle-income countries: A systematic review. Vaccine. (2024) 42:1200–10. doi: 10.1016/j.vaccine.2024.01.094, 38302338 PMC10911079

[44] Mvundura M Slavkovsky R Debellut F Naddumba T Bayeh A Ndiaye C . Cost and operational context for national human papillomavirus (HPV) vaccine delivery in six low- and middle-income countries. Vaccine. (2023) 41:7435–43. doi: 10.1016/j.vaccine.2023.11.008, 37949752 PMC10697825

[45] World Health Organization. WHO position papers on Human papillomavirus (HPV). Geneva: WHO; 2022 [updated 2024 Oct 3]. Available online at: https://www.who.int/teams/immunization-vaccines-and-biologicals/policies/position-papers/human-papillomavirus-%28hpv%29 (accessed October 25, 2025).

[46] World Health Organization. WHO adds an HPV vaccine for single-dose use. Geneva: WHO; 2024. Available online at: https://www.who.int/news/item/04-10-2024-who-adds-an-hpv-vaccine-for-single-dose-use (accessed October 25, 2025).

[47] United Nations Children’s Fund (UNICEF) Supply Division. Human Papilloma Virus (HPV) vaccine price data. Copenhagen: UNICEF; [updated 2025 Sep 25]. (2025). Available online at: https://www.unicef.org/supply/documents/human-papilloma-virus-hpv-vaccine-price-data (accessed September 25, 2025).

[48] Pan American Health Organization (PAHO). PAHO revolving fund: Prices for the calendar year 2025. Washington, DC: PAHO; 2025. Available online at: https://www.paho.org/sites/default/files/2025-07/prices-vac-2025-eng-2-july-2025-v3.pdf (accessed October 25, 2025).

[49] Gavi, the Vaccine Alliance. Co-financing policy. Geneva: Gavi, the Vaccine Alliance; 2025. Available online at: https://www.gavi.org/programmes-impact/programmatic-policies/co-financing-policy (accessed October 25, 2025).

[50] Gavi, the Vaccine Alliance. Eligibility & co-financing policies. Geneva: Gavi, the Vaccine Alliance; 2025. Available online at: https://www.gavi.org/programmes-impact/programmatic-policies/eligibility-co-financing-policies (accessed October 25, 2025).

[51] World Health Organization. WHO updates recommendations on HPV vaccination schedule. Geneva: WHO; 2022. Available online at: https://www.who.int/news/item/20-12-2022-WHO-updates-recommendations-on-HPV-vaccination-schedule (accessed October 25, 2025).

[52] Graham DM Isaranuwatchai W Habbous S de Oliveira C Liu G Siu LL . A cost-effectiveness analysis of human papillomavirus vaccination of boys for the prevention of oropharyngeal cancer. Cancer. (2015) 121:1785–92. doi: 10.1002/cncr.29111, 25867018

[53] World Health Organization. Behavioural and social drivers of vaccination: Tools and practical guidance for achieving high uptake. Geneva: WHO; 2022. Available online at: https://www.who.int/publications/i/item/9789240049680 (accessed October 25, 2025).

[54] United Nations Children’s Fund (UNICEF). Vaccine messaging guide: A handbook for delivering key messages on vaccine equity and access. Copenhagen: UNICEF; 2023. Available online at: https://www.unicef.org/media/138031/file/Vaccine%20Messaging%20Guide.pdf (accessed October 25, 2025).

[55] World Health Organization. Implementing the immunization agenda 2030: A framework for action through coordinated planning, monitoring & evaluation, Ownership & Accountability, and Communications & Advocacy. Geneva: WHO; 2020 [version v04]. Available online at: https://www.immunizationagenda2030.org/images/documents/IA2030_FrameworkForActionv04.pdf (accessed October 25, 2025). 10.1016/j.vaccine.2021.09.045 PMC10801759 36639274

[56] Centers for Disease Control and Prevention. Immunization information systems (IIS) resources: About IIS. Atlanta: CDC; 2024. Available online at: https://www.cdc.gov/iis/about/index.html (accessed October 25, 2025).

[57] Centers for Disease Control and Prevention. Rapid community assessment guide: A guide to help you understand your community’s needs around vaccination. Atlanta: CDC; 2023. Available online at: https://www.cdc.gov/vaccines/php/downloads/Rapid-Community-Assessment-Guide_2023.pdf (accessed October 25, 2025).

[58] Public Health Agency of Canada. Canadian adverse events following immunization surveillance system (CAEFISS). Ottawa: PHAC; 2025 [updated 2025 Feb 4]. Available online at: https://www.canada.ca/en/public-health/services/immunization/canadian-adverse-events-following-immunization-surveillance-system-caefiss.html (accessed October 25, 2025).

[59] Binagwaho A Wagner CM Gatera M Karema C Nutt CT Ngabo F. Achieving high coverage in Rwanda's national human papillomavirus vaccination programme. Bull World Health Organ. (2012) 90:623–8. doi: 10.2471/BLT.11.097253, 22893746 PMC3417784

[60] Dorji T Tshomo U Phuntsho S Tamang TD Tshokey T Baussano I . Introduction of a national HPV vaccination program into Bhutan. Vaccine. (2015) 33:3726–30. doi: 10.1016/j.vaccine.2015.05.078, 26057136

[61] Baussano I Sayinzoga F Tshomo U Tenet V Vorsters A Heideman DAM . Impact of human papillomavirus vaccination, Rwanda and Bhutan. Emerg Infect Dis. (2021) 27:1–9. doi: 10.3201/eid2701.191364, 33350922 PMC7774553

[62] World Health Organization. Data quality assurance: Module 3 – Site assessment of data quality: Data verification and system assessment. Geneva: WHO; 2020. Available online at: https://cdn.who.int/media/docs/default-source/data-quality-pages/2021_dqa_module-3_site-assessment-framework.pdf (accessed October 24, 2025).

[63] Centers for Disease Control and Prevention. IIS functional standards introduction. Atlanta: CDC; 2025 [updated 2024 Apr 22]. Available online at: https://www.cdc.gov/iis/functional-standards/introduction.html (accessed October 25, 2025).

[64] Centers for Disease Control and Prevention. Immunization information system functional standards version 4.1 resource guide. Atlanta: CDC; 2022. Available online at: https://www.cdc.gov/iis/downloads/func-stds-v4-1-resource-guide.pdf (accessed October 24, 2025).

