# Supplementary material for: Gender-neutral human papillomavirus vaccination: an equitable and cost-effective public health investment
Source: Front Public Health. 2026 Jan 5;13:1725127. doi: 10.3389/fpubh.2025.1725127 (PMC12813166; doi:10.3389/fpubh.2025.1725127)
Supplement: Supplementary file 1 [file Table_1.DOCX]

**Supplementary Table S1. Summary of cost-effectiveness (ICER) estimates for gender-neutral HPV vaccination (selected settings).**

| **Country/setting** | **Perspective** | **Comparator** | **Coverage & horizon** | **Key modeled outcomes** | **Summary result** |
| --- | --- | --- | --- | --- | --- |
| Japan (HIC) | Payer & societal | Gender-neutral 9vHPV vs female-only | F 30%, M 15%; 100-y horizon | +2,070 female and +1,773 male cancer deaths averted | ICER JPY 4,798,537 per QALY (≈USD 33,000) (payer); JPY 4,248,586 per QALY (≈USD 29,000) (societal); cost-effective at low coverage [34] |
| Singapore (HIC) | Healthcare | Gender-neutral (bi-/9v) vs status quo | 80% 13-year-olds; lifetime | 30–34 HPV-related cancers averted per cohort | Likely cost-effective at 1.5% discount (bivalent: SGD 19,007 per QALY [≈USD 14,000] ); not cost-effective at 3% [38] |
| South Africa (UMIC) | Health system (DALYs) | Gender-neutral vs girls-only | 80% GNV; 2024–2120 | Similar incidence impact to 90% girls-only; threshold pricing assessed | USD 2,782 per DALY averted vs girls-only; 9vHPV cost-effective if unit price < USD 40 per dose [41] |
| Colombia (UMIC) | Healthcare | Gender-neutral 4vHPV vs female-only (35% VCR) | 100-year horizon | Large reductions in HPV-6/11-driven disease (warts) | Cost-saving (ICER < 0) vs 35%-coverage girls-only; net savings USD 88–184 million over 100 years [42] |

**Notes.** ICER values reflect each model’s assumptions regarding discount rates, vaccine prices, screening background, sexual network structure, time horizon, and outcome set. Because these vary across settings, ICERs are not directly comparable. Where reported, net savings are shown. Abbreviations: GNV = gender-neutral vaccination; QALY = quality-adjusted life-year; DALY = disability-adjusted life-year; VCR = vaccination coverage rate; F/M = female/male; 2v/9v = bivalent/9-valent HPV vaccine. Abbreviations and comparators follow the cited sources [34,38,41,42].
