# Supplementary material for: Gender-neutral human papillomavirus vaccination: an equitable and cost-effective public health investment
Source: Front Public Health. 2026 Jan 5;13:1725127. doi: 10.3389/fpubh.2025.1725127 (PMC12813166; doi:10.3389/fpubh.2025.1725127)
Supplement: Supplementary file 2 [file Table_2.DOCX]

| **Component** | **Change with gender-neutral expansion** | **Budget signal / practical lever** |
| --- | --- | --- |
| Commodities | Doses roughly double under 2-dose rules; one-dose policy cuts needs by ~50% for most 9–20-year-olds, where permitted by WHO (except immunocompromised) | Use pooled price files (PAHO Revolving Fund; UNICEF Supply Division) for scenario planning; state price-year explicitly; prioritize single-dose where aligned with WHO [48,51] |
| Procurement | Larger volumes enable firmer, multi-year awards | Lock in multi-year framework awards via PAHO/UNICEF; use published HPV price files for volume/commitment planning [47] |
| Financing / cost-sharing | For Gavi-eligible countries, incremental doses co-financed and phased to domestic budgets | Cite Gavi co-financing policy in budget notes and plan a glidepath to full domestic financing as applicable [49] |
| Delivery platform | School sessions add boys in the same roster/consent flow; route plans unchanged | Delivery overhead typically marginal when school-based; track sex-disaggregated coverage to adjust session counts (operational KPI suite as in Table 2). |
| Sensitivity levers | Vaccine price; discount rate; coverage; schedule (1 vs 2 doses) | - Run low/high unit-price and one-dose scenarios; present country-specific price thresholds as scenario tests (e.g., <USD 40 per 9v dose in the South Africa analysis) [41]. |

**Supplementary Table S2. Incremental budget and operational impacts when expanding from girls-only to gender-neutral HPV vaccination using existing school platforms.**

*Notes:* **GNV** = gender-neutral vaccination; **PAHO** = Pan American Health Organization; **UNICEF** = United Nations Children’s Fund. “One-dose” refers to WHO 2022 schedule flexibility for most 9–20-year-olds (except immunocompromised). Use current pooled price files (PAHO Revolving Fund; UNICEF Supply Division) and specify **price-year** in scenarios. In most LIC/LMIC/UMIC settings with school platforms, the marginal fiscal signal is primarily **commodity-driven** rather than delivery-driven; pairing single-dose schedules, pooled procurement, and staged co-financing keeps expansion affordable and plannable over multi-year horizons [41,47–49,51].
*Illustrative 2025 pooled prices (for transparency, optional):* HPV2 USD 2.90; HPV4 USD 10.48; HPV9 USD 14.99 [47,51].
