# Supplementary material for: Gender-neutral human papillomavirus vaccination: an equitable and cost-effective public health investment
Source: Front Public Health. 2026 Jan 5;13:1725127. doi: 10.3389/fpubh.2025.1725127 (PMC12813166; doi:10.3389/fpubh.2025.1725127)
Supplement: Supplementary file 3 [file Table_3.DOCX]

**Supplementary Table S3. Evidence sources informing this policy-oriented Perspective**

| **Source category** | **Sources consulted** | **Rationale (why these sources)** | **Last searched (date)** | **Main topics covered** | **How evidence was prioritized (brief)** |
| --- | --- | --- | --- | --- | --- |
| Peer-reviewed literature | PubMed/MEDLINE | Broad biomedical and public health coverage; efficient retrieval of vaccine schedule, implementation, and economic evaluation literature | 25 Oct 2025 | One-dose/single-dose HPV schedules; gender-neutral vaccination; priority groups (e.g., MSM); implementation strategies; safety surveillance; economic considerations | Prioritized guidelines/position papers, systematic reviews, and high-quality empirical studies with direct policy/program relevance; de-prioritized studies without program implications |
| Global policy & technical guidance | World Health Organization (WHO) position papers and program guidance | Primary source for global recommendations (schedule options, eligibility, implementation considerations) | 25 Oct 2025 | Schedule flexibility (including one-dose); gender-neutral vaccination considerations; safety monitoring expectations | Treated as primary policy anchors; interpreted in light of feasibility, equity, and local epidemiology/economics |
| Procurement / market information | UNICEF procurement information (e.g., awarded prices); PAHO price lists | Supports budgeting feasibility and pooled procurement considerations in resource-constrained settings | 25 Oct 2025 | Multi-year budgeting; pooled procurement; price visibility | Used to contextualize budgeting and procurement feasibility; not used as effectiveness/safety evidence |
| Hand-searching | Reference lists of key guidelines/reviews and highly relevant policy papers | Captures program-relevant items not easily retrieved via keyword searches | 25 Oct 2025 | Implementation details; case examples; monitoring indicators | Included when directly informative for program design or monitoring |
| National / regional immunization guidance | National Immunization Technical Advisory Group (NITAG) statements and national guidelines (e.g., ACIP/JCVI/ECDC—include only those cited). | Reflect real-world program decisions and implementation constraints beyond global guidance. | 25 Oct 2025 | Gender-neutral policy decisions; MSM-targeted strategies; catch-up and age eligibility; delivery models. | Used as contextual implementation evidence; preference to the most recent/updated statements and those aligned with cited program data. |
| Safety surveillance systems / AEFI reporting | National AEFI surveillance summaries and program safety reports (e.g., Canada’s AEFI surveillance system if cited; WHO safety guidance). | Support statements on safety monitoring expectations and reporting practice. | 25 Oct 2025 | AEFI definitions/reporting; safety signal monitoring; dashboard/registry linkage concepts. | Used for surveillance approach and definitions; not used to adjudicate causality beyond what official assessments report. |

**Footnote (optional):** Key terms combined variants of: HPV vaccine/vaccination, one-dose/single-dose, gender-neutral, boys/male, MSM, equity/priority populations, coverage/uptake, catch-up, cost-effectiveness/economic evaluation, procurement/price, safety/AEFI, surveillance/monitoring. This Perspective used an evidence-informed narrative synthesis (not a PRISMA systematic review): inclusion focused on policy- and program-relevant human evidence (guidelines, systematic reviews, RCTs, large observational studies, and explicit economic/transmission models). For included sources, we extracted setting/population, schedule/delivery features, endpoints (coverage/equity/safety/economic implications), and implementation constraints, and mapped findings to the recommendation domains. “No quantitative pooling or formal risk-of-bias grading was performed, consistent with a policy-oriented Perspective.”
