# Supplementary material for: Gender-neutral human papillomavirus vaccination: an equitable and cost-effective public health investment
Source: Front Public Health. 2026 Jan 5;13:1725127. doi: 10.3389/fpubh.2025.1725127 (PMC12813166; doi:10.3389/fpubh.2025.1725127)
Supplement: Supplementary file 4 [file Table_4.DOCX]

**Supplementary Methods — Operational Specifications for School-based Gender-neutral HPV Program**

**Alignment statement.**
These program-defined indicators and conventions are **aligned** with WHO AEFI causality guidance and Brighton case definitions, WHO *Immunization in Practice* (IIP), RED microplanning, WHO Data Quality Review (DQR), and CDC IIS Functional Standards. **Numeric thresholds (e.g., 48 h, 30/60 d, 95%/98%) are program-set** for management rather than mandated by guidelines.

**Abbreviations.** AEFI, adverse event following immunization; DQR, Data Quality Review; IIS, immunization information system; KPI, key performance indicator; RED, Reaching Every District.

**S1. AEFI reporting & response**

**S1.1 Purpose & scope**

Define a pragmatic safety surveillance package that (i) yields stable rates for routine review, (ii) assures timely investigation of **serious** AEFIs, and (iii) feeds transparent **quarterly** safety briefs (aligned with WHO/CIOMS good-practice communication).

**S1.2 Indicators (definitions & computation)**

**KPI-S1a. AEFI reporting rate (per 100,000 doses)**

- **Numerator:** All AEFI reports that meet the **national** case definition (any severity) within the window; where national guidance is silent, adopt **WHO/Brighton-aligned** definitions.
- **Denominator:** Total HPV doses administered in the same window (IIS).
- **Formula:** 100000 × AEFI_reports / doses_administered.
- **Window:** Monthly; summarize quarterly/annually.
- **Disaggregation:** sex; age bands (e.g., 9–10, 11–12, 13–15, ≥16); district/region; product/lot; dose (D1/D2).
- **Use:** Stability (run charts/SPC); lot clustering screen.

**KPI-S1b. Serious AEFI investigation timeliness (48 h start)**

- **Numerator:** Serious AEFIs with investigation **initiated ≤48 h** of notification.
- **Denominator:** All serious AEFIs notified in the window.
- **Target (program-set):** ≥95% initiated ≤48 h.

**KPI-S1c. Causality assessment completion (30/60 d)**

- **Numerator:** AEFIs (serious + selected signals) with documented causality **completed ≤30 d** (primary) and **≤60 d** (secondary).
- **Denominator:** AEFIs requiring causality assessment.
- **Targets (program-set):** ≥90% (30 d); ≥98% (60 d).

**KPI-S1d. Public reporting cadence**

- **Definition:** **Quarterly** safety bulletin with counts, rates, timeliness, plain-language narrative.
- **Target:** 100% of quarters covered; ≤45 d lag from quarter-end.
- *(Optional national exemplar to cite in text: CAEFISS.)*

**Case definitions.** Use national definitions (e.g., anaphylaxis, syncope, fever, injection-site reactions). Where absent, apply WHO/Brighton definitions internally and reconcile with national systems when available.

**S1.3 Minimum data elements (AEFI case form/register)**

De-identified patient ID; sex; DOB/age; school & district; vaccination date/time; **vaccine product/valency/manufacturer/lot/dose**; onset date/time; signs/symptoms; seriousness (Y/N + rationale); hospitalization (Y/N); initial management; reporter name/role/contact; notification date/time; investigation **start** date/time; investigation outcome; **causality category**; final outcome; remarks; IIS linkage.

**S1.4 Reporting tables (shells)**

**Table S1-A. Monthly AEFI summary (rates per 100,000 doses)**

| Month | Doses | All AEFI (n / rate) | Serious (n / rate) | 48 h start % | 30 d causality % | Lots flagged (n) |
| --- | --- | --- | --- | --- | --- | --- |

**Table S1-B. Disaggregation by sex & district (quarter)**

| District | Doses | AEFI n | Rate/100k | Serious n | 48 h start % | Notes |
| --- | --- | --- | --- | --- | --- | --- |

*Optional:* “Top 5 lots by AEFI rate” screen with alert thresholds.

**S1.5 Edge cases & rules**

- Multiple symptoms within the **same episode** = **one** AEFI report.
- Onset within window but dose outside → count in **onset** window; align denominator to doses in the **same** window.
- Merge duplicate reports (same patient/episode) with audit trail.
- For multi-product programs, publish **product-specific** rates.

**S1.6 Example computations (pseudo-SQL)**

-- Denominator

SELECT date_trunc('month', dose_datetime) AS month, COUNT(*) AS doses

INTO temp_doses

FROM vacc

WHERE vaccine='HPV'

GROUP BY 1;

-- All AEFI (any severity) by onset

SELECT date_trunc('month', onset_datetime) AS month, COUNT(*) AS aefi_all

INTO temp_aefi

FROM aefi

WHERE case_meets_definition = TRUE

GROUP BY 1;

-- Serious AEFI timeliness (48h from notification to investigation start)

SELECT date_trunc('month', notify_datetime) AS month,

COUNT(*) FILTER (WHERE serious = TRUE) AS serious_total,

COUNT(*) FILTER (

WHERE serious = TRUE

AND investigation_start <= notify_datetime + INTERVAL '48 hours'

) AS serious_48h

INTO temp_timeliness

FROM aefi

GROUP BY 1;

-- Join and compute outputs

SELECT d.month,

d.doses,

a.aefi_all,

100000.0 * a.aefi_all / d.doses AS rate_all_per100k,

t.serious_total,

100000.0 * t.serious_total / d.doses AS serious_rate_per100k,

100.0 * t.serious_48h / NULLIF(t.serious_total,0) AS pct_48h

FROM temp_doses d

LEFT JOIN temp_aefi a USING(month)

LEFT JOIN temp_timeliness t USING(month)

ORDER BY d.month;

**S1.7 Communication & transparency**

Monthly internal dashboards; **quarterly public brief**; pre-approved templates for addressing false signals (background rates, coincidental events). De-identify all outputs; retain lot numbers internally for actioning.

**S2. Session execution & supply reliability**

**S2.1 Purpose & scope**

Track delivery performance to ensure high session availability and **reliable cold chain**; use measures to trigger rescheduling, buffer adjustments, and field support (consistent with IIP/RED/DQR/IIS).

**S2.2 Indicators (definitions & computation)**

**KPI-S2a. Planned sessions conducted**

- **Numerator:** Sessions **held** within the window.
- **Denominator:** Sessions **planned** for the same window (micro-plans/calendars).
- **Rules:** “Held” includes sessions with **zero eligible attendees** (record reason). Sessions **postponed ≤7 d** count as held in the **rescheduled** window; track postponement rate separately.
- **Window:** Term/campaign (and monthly).
- **Target (program-set):** ≥95% sessions conducted.

**KPI-S2b. Stockout-free session-days**

- **Numerator:** Session-days **without stockout** of HPV vaccine **or** critical consumables (syringes, safety boxes) during planned hours.
- **Denominator:** All session-days conducted.
- **Target (program-set):** ≥98% stockout-free.

**KPI-S2c. Cold-chain excursions investigated**

- **Definition:** Share of sessions with a documented **temperature excursion** (<2 °C or >8 °C, or logger alarm) with investigation **initiated ≤24 h** and disposition (use/quarantine/discard) **documented ≤72 h**.
- **Targets (program-set):** ≥95% initiation ≤24 h; ≥95% disposition ≤72 h.
- **Display:** Excursions per **100 session-days**.

**Optional (program-set; show in Supplement only):**

- **Wastage rate** = [(doses issued − doses administered − closing balance) / doses issued] × 100%.
- **Reschedule timeliness** = sessions postponed that were delivered **≤7 d** / all postponed.
- **Staffing reliability** = sessions staffed as planned / sessions planned.

**S2.3 Minimum data elements**

**Session register:** school ID & name; district; planned date; start–end time; **held? (Y/N)**; rescheduled date; **doses at start**; **doses used**; closing balance; **stockout occurrence** (Y/N; item; start–end time); **cold-chain status** (logger ID, min/max, alarm Y/N); excursion investigation start/end; reason for cancellation/postponement; staff present; remarks.
**Stock ledger (summary extract):** opening; receipts; issues to session; returns; closing; wastage (open/closed vial); lot numbers.

**S2.4 Reporting tables (shells)**

**Table S2-A. Session execution (by district, term)**

| District | Sessions planned | Held | % held | Postponed | Cancelled | Rescheduled ≤7 d % | Notes |
| --- | --- | --- | --- | --- | --- | --- | --- |

**Table S2-B. Supply reliability (by month)**

| Month | Session-days | Stockout-free n | Stockout-free % | Cold-chain excursions (n / per 100 session-days) | 24 h investigation % | 72 h disposition % |
| --- | --- | --- | --- | --- | --- | --- |

*Root-cause codes (examples):* supply delay; distribution planning error; staff absence; school closure; extreme weather; consent delay; data issue (duplicate roster); other (specify).

**S2.5 Edge cases & rules**

- Planned but zero attendance → **count as held** with reason “0 eligible/absent” (avoids inflated cancellations).
- **Partial stockout** (e.g., syringes only) still counts as stockout; record duration and any turned-away clients.
- **Transport alarms ≤24 h** before session for intended supplies → attribute to that **session-day**.
- Integrated sessions (HPV + others): compute **HPV-specific** stockout; record other vaccines separately.

**S2.6 Example computations (pseudo-SQL)**

-- Planned vs held

SELECT term, district,

COUNT(*) FILTER (WHERE planned=TRUE) AS sessions_planned,

COUNT(*) FILTER (WHERE held=TRUE) AS sessions_held,

ROUND(

100.0 * COUNT(*) FILTER (WHERE held=TRUE) /

NULLIF(COUNT(*) FILTER (WHERE planned=TRUE),0), 1

) AS pct_held

FROM sessions

GROUP BY term, district;

-- Stockout-free session-days

SELECT date_trunc('month', session_date) AS month,

COUNT(*) AS session_days,

COUNT(*) FILTER (WHERE stockout=FALSE) AS stockout_free,

ROUND(100.0 * COUNT(*) FILTER (WHERE stockout=FALSE) / COUNT(*), 1) AS pct_stockout_free

FROM sessions

WHERE held=TRUE

GROUP BY 1;

-- Cold-chain excursion follow-up timeliness

SELECT date_trunc('month', session_date) AS month,

COUNT(*) FILTER (WHERE cc_excursion=TRUE) AS excursions,

100.0 * COUNT(*) FILTER (

WHERE cc_excursion=TRUE AND investigation_start <= session_date + INTERVAL '24 hours'

) / NULLIF(COUNT(*) FILTER (WHERE cc_excursion=TRUE),0) AS pct_24h_investigation,

100.0 * COUNT(*) FILTER (

WHERE cc_excursion=TRUE AND disposition_time <= investigation_start + INTERVAL '72 hours'

) / NULLIF(COUNT(*) FILTER (WHERE cc_excursion=TRUE),0) AS pct_72h_disposition

FROM sessions

GROUP BY 1;

**S2.7 Action thresholds & management**

- **% held <95%** → supportive supervision; review micro-plans & consent workflow.
- **Stockout-free <98%** → review forecasting, distribution timing, buffer stock rules.
- **Excursion clusters** → route audit; retraining on logger handling; lot disposition per policy.
- **Monthly** district reviews; **quarterly** national summary with corrective actions.
